# Water Deficit and Heat Affect the Tolerance to High Illumination in Hibiscus Plants

**DOI:** 10.3390/ijms14035432

**Published:** 2013-03-07

**Authors:** Romualdo Muñoz, María José Quiles

**Affiliations:** Department of Plant Biology, University of Murcia, 30100 Espinardo Murcia, Spain; E-Mail: rmunoz@um.es

**Keywords:** chlororespiration, NDH complex, *Hibiscus rosa-sinensis*, PGR5, PTOX

## Abstract

This work studies the effects of water deficit and heat, as well as the involvement of chlororespiration and the ferredoxin-mediated cyclic pathway, on the tolerance of photosynthesis to high light intensity in *Hibiscus rosa-sinensis* plants. Drought and heat resulted in the down–regulation of photosynthetic linear electron transport in the leaves, although only a slight decrease in variable fluorescence (F_v_)/maximal fluorescence (F_m_) was observed, indicating that the chloroplast was protected by mechanisms that dissipate excess excitation energy to prevent damage to the photosynthetic apparatus. The incubation of leaves from unstressed plants under high light intensity resulted in an increase of the activity of electron donation by nicotinamide adenine dinucleotide phosphate (NADPH) and ferredoxin to plastoquinone, but no increase was observed in plants exposed to water deficit, suggesting that cyclic electron transport was stimulated by high light only in control plants. In contrast, the activities of the chlororespiration enzymes (NADH dehydrogenase (NDH) complex and plastid terminal oxidase (PTOX)) increased after incubation under high light intensity in leaves of the water deficit plants, but not in control plants, suggesting that chlororespiration was stimulated in stressed plants. The results indicate that the relative importance of chlororespiration and the cyclic electron pathway in the tolerance of photosynthesis to high illumination differs under stress conditions. When plants were not subjected to stress, the contribution of chlororespiration to photosynthetic electron flow regulation was not relevant, and another pathway, such as the ferredoxin-mediated cyclic pathway, was more important. However, when plants were subjected to water deficit and heat, chlororespiration was probably essential.

Abbreviations

DW: dry weight
ETR: relative electron transport rate
F_m_: maximal fluorescence yield in the dark adapted state
F_0_: minimal fluorescence yield in the dark adapted state
F_v_: variable fluorescence
FW: fresh weight
NDH: NADH dehydrogenase
NADH-PQR: NADH-plastoquinone oxidoreductase
PAM: pulse amplitude modulation
PPFD: photosynthetic photon flux density
PS: photosystem
PTOX: plastid terminal oxidase
ROS: reactive oxygen species
RWC: relative water content
TW: turgid weight

## 1. Introduction

Environmental stresses, such as high irradiance, high temperature and drought, negatively affect photosynthetic processes [1–3]. Plants are frequently exposed to stress, both in natural and agricultural conditions, and such exposure plays a major role in determining the distribution of plant species across different types of environment and in limiting crop productivity. Understanding the physiological processes that underlie stress injury and the tolerance mechanisms of plants to environmental stress is of immense importance to both agriculture and the environment.

Tolerance to stress results from integrated events occurring at all organization levels, from anatomical and morphological, to cellular, biochemical and molecular levels. At the biochemical level, plants alter their metabolism in various ways to accommodate environmental stress, and photosynthesis is one of these ways.

Photosynthesis in chloroplasts involves a vectorial electron transfer from water in the lumen to nicotinamide adenine dinucleotide phosphate (NADP^+^) in the stroma, by means of redox carriers. Besides this major pathway, alternative electron transfer pathways, involving non-photochemical reduction or the oxidation of plastoquinones at the expense of stromal electron donors or acceptors, have been proposed based on functional measurements. These additional reactions cover two main concepts, one based on the cycling of electrons around PS I [4–6] and the other on chlororespiration [7,8], which consists of electron transfer reactions from stromal reductants to O_2_ through the plastoquinone pool [5,9,10].

Two thylakoidal enzymes, both of which are important in chlororespiration, have been molecularly characterized: the plastid-encoded NADH dehydrogenase (NDH) complex [11–16] and the nucleus-encoded plastid-localized terminal oxidase (PTOX) [17–19]. The NDH complex is an entry point for electrons into the photosynthetic electron-transport chain, involving the non-photochemical reduction of plastoquinones and PTOX as a point of electron transfer from plastoquinol to molecular oxygen, resulting in the formation of water in the stroma and reducing the formation of reactive oxygen species [9]. In addition to chlororespiration, the NDH complex is involved in the cyclic electron flow around PS I [20]. Two parallel cyclic pathways exist around PS I [20], one involving the NDH complex and the other sensitive to antimycin A, in which two proteins are essential components: the thylakoid membrane protein encoded by the *pgr5* gene (PGR5) and the thylakoid transmembrane protein (PGRL1), which interacts functionally and physically with PGR5 [21–23]. The physiological role of the chloroplast electron pathways operating around PS I has been difficult to establish. Although these reactions probably do not play a major role during photosynthesis under optimal conditions [24–27], they probably participate in the flexibility of electron transfer reactions required to balance ATP/NADPH requirements when photosynthesis operates under changing environmental conditions [10,28–33].

Several studies have proposed that chlororespiratory components may be involved in the protective or adaptive mechanisms of plants in response to environmental stress, such as heat, water deficit and high light [17,34–45]. Recently, we described the increase in the NDH complex and PTOX in *Spathiphyllum wallisii*, a shade species very sensitive to heat and high illumination, when the above mentioned stresses were combined with water deficit [44]. Additionally, the involvement of the NDH-mediated cyclic electron pathways around PS I in supplying the extra adenosine triphosphate (ATP) required in conditions of mild water stress has been suggested [10]. These results suggested that the NDH complex and PTOX are heat and water stress dependent. However, the cooperation of the cyclic electron pathways and chlororespiration, under stress conditions, in photosynthesis remains unclear [11]. Additional experiments are needed to elucidate the relative importance of these pathways in the tolerance of photosynthesis to high illumination under stress caused by drought. The present work studies the effects of water deficit and heat, as well as the involvement of chlororespiratory enzymes and ferredoxin-mediated cyclic electron flow on the tolerance of photosynthesis to high light intensity in *Hibiscus rosa-sinensis* plants.

## 2. Results

### 2.1. Changes in the Relative Water Content and in Fluorescence Parameters

**Figure 1:**
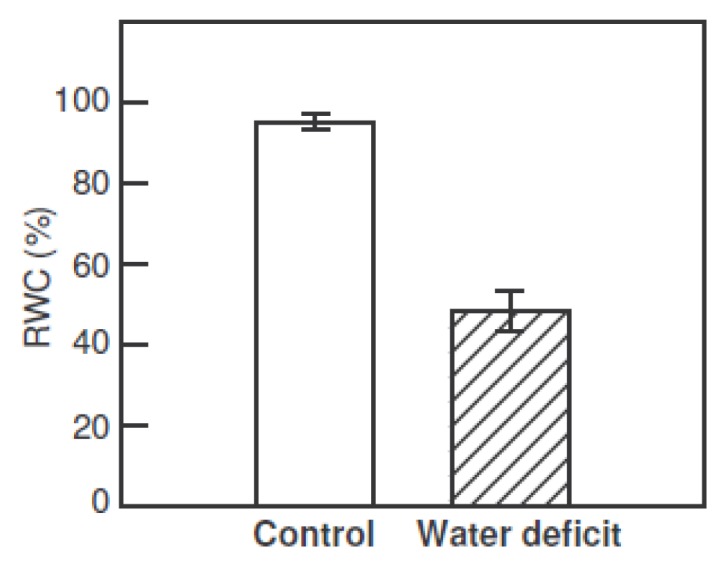
The relative water content (RWC) of leaves from *H. rosa-sinensis* plants at the start of the experiment (control) and after exposure to three photoperiods (18 h, 60 μmol·m^−2^·s^−1^ photosynthetic photon flux density (PFFD) and 35 °C) with reduced irrigation (water deficit). The stress photoperiods were separated by 6 h night-periods at 24 °C. The values are the means ± SE from four independent replicates.

Plant water status was estimated by measuring the relative water content (RWC) of leaves in plants at the start of the experiment (control) and immediately after exposure to three photoperiods with reduced irrigation and heat (Figure 1). The RWC decreased to around 50% after three stress photoperiods with low watering levels.

**Figure 2:**
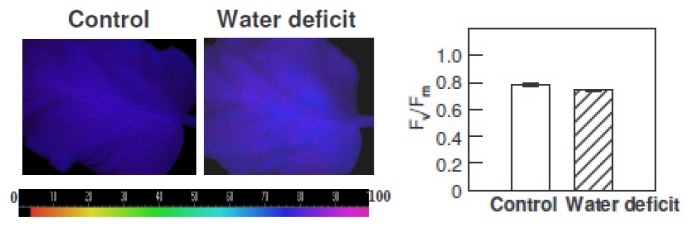
Images of the maximal quantum yield of PS II (variable fluorescence (F_v_)/maximal fluorescence (F_m_)) from a typical leaf of *H. rosa-sinensis* plants in control conditions and after exposure to three photoperiods (18 h, 60 μmol·m^−2^·s^−1^ PPFD and 35 °C) with reduced irrigation (water deficit). The stress photoperiods were separated by 6 h night-periods at 24 °C. The images are color-coded according to the pattern (0 to 1 × 100 range) shown below the images. The figure shows representative images from four independent experiments. The histograms show the means ± SE from four independent replicates.

The fluorescence imaging technique was used to assess the maximal quantum yield of PS II (variable fluorescence (F_v_)/maximal fluorescence (F_m_)) in intact leaves from plants in control conditions and when exposed to three photoperiods with reduced irrigation and heat (Figure 2). The results are shown as color-coded images and as histograms of the mean F_v_/Fm values ± SE. After the three stress photoperiods, the F_v_/F_m_ decreased by around 5%.

**Figure 3:**
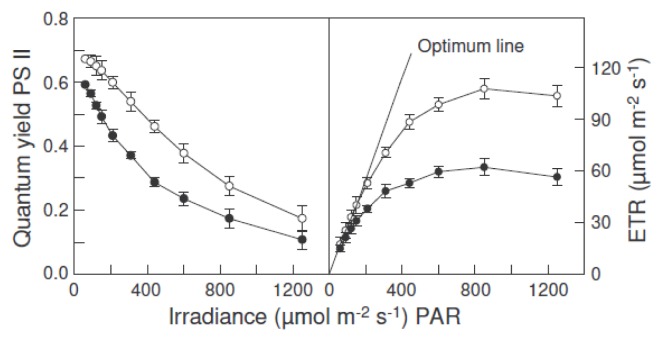
Light response curves for the quantum yield of PS II and the relative electron transport rate (ETR) in intact dark adapted leaves from *Hibiscus rosa-sinensis* plants in control conditions (white circles) and after exposure to three photoperiods (18 h, 60 μmol·m^−2^·s^−1^ PPFD and 35 °C) with reduced irrigation (black circles). The stress photoperiods were separated by 6 h night-periods at 24 °C. The values are means ± SE from five independent replicates.

The light response curves for the quantum yield of PS II and the relative electron transport rate in intact leaves from control plants and plants exposed to water deficit and heat were studied using the fluorescence imaging technique (Figure 3). The PS II quantum yields and the relative electron transport rates decreased (by approximately 39% and 42%, respectively) in plants exposed to water deficit and heat.

### 2.2. Electron Donation to Plastoquinone in Osmotically Ruptured Chloroplasts

**Figure 4:**
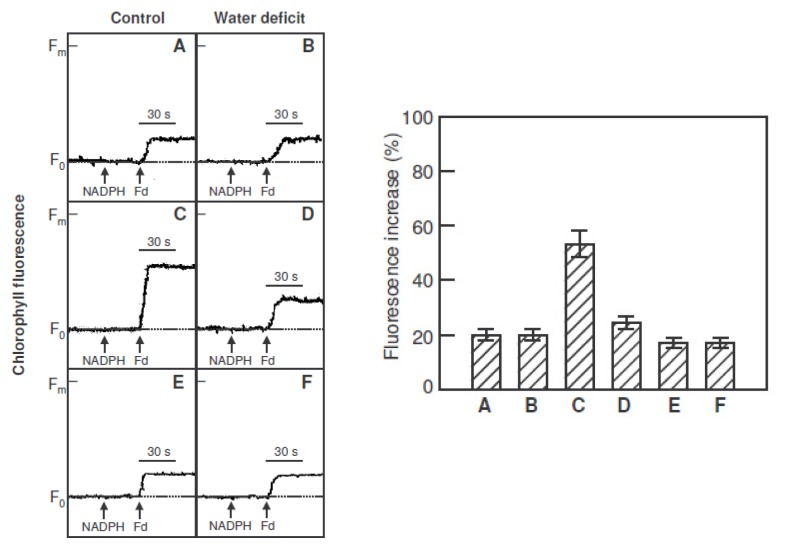
Increases in chlorophyll fluorescence after addition of nicotinamide adenine dinucleotide phosphate (NADPH) (0.25 mM) 30 s into the run and ferredoxin (Fd, 5 μM) 60 s into the run, under weak measuring light (1.0 μmol·m^−2^·s^−1^) in thylakoid membranes (50 μg Chl·mL^−1^) isolated from recently detached leaves (**A** and **B**) or leaves incubated in water for 20 h under high light intensity (1400 μmol·m^−2^·s^−1^) at 24 °C (**C** and **D**). The leaves were detached from *H. rosa-sinensis* plants in control conditions and after exposure to three photoperiods (18 h, 60 μmol·m^−2^·s^−1^) PPFD and 35 °C with reduced irrigation (water deficit). The stress photoperiods were separated by 6 h night-periods at 24 °C. Thylakoid suspensions from leaves incubated in water for 20 h were additionally incubated with antimycin A (2 μM) for 2 min prior to the measurements (**E** and **F**). The figure shows typical curves and the histograms of the means ± SE from five independent replicates.

The activity of electron donation by NADPH and ferredoxin to plastoquinone was assayed as an increase in the chlorophyll fluorescence emitted during exposure to light of a very low intensity (1.0 μmol m^−2^ s^−1^). The fluorescence level reflects the reduction of plastoquinone by electron transport from ferredoxin [21]. Chlorophyll fluorescence increased after the addition of NADPH (0.25 mM) and ferredoxin (5 μM) under weak measuring light (Figure 4) in thylakoid membrane suspensions (50 μg Chl mL^−1^) isolated from chloroplasts of leaves recently detached (Figure 4A,B) or incubated in water for 20 h under high light intensity (1400 μmol m^−2^ s^−1^) at 24 °C (Figure 4C,D). Thylakoid suspensions from leaves incubated in water for 20 h were additionally incubated with antimycin A (2 μM) for 2 min prior to the measurements (Figure 4E,F). Control leaves incubated under high light intensity showed a greater increase of chlorophyll fluorescence (53%) than control leaves recently detached (20%). However, leaves from plants exposed to water deficit and heat, both recently detached and incubated under high light intensity, showed similar chlorophyll fluorescence levels (20% and 23%, respectively). Antimycin A inhibited the increase of chlorophyll fluorescence compared with measurements made without inhibitor, although its effect on leaves from plants exposed to water deficit and heat was slight (Figure 4F).

### 2.3. PTOX and NADH-PQR Activities in the Thylakoid Membranes

**Figure 5:**
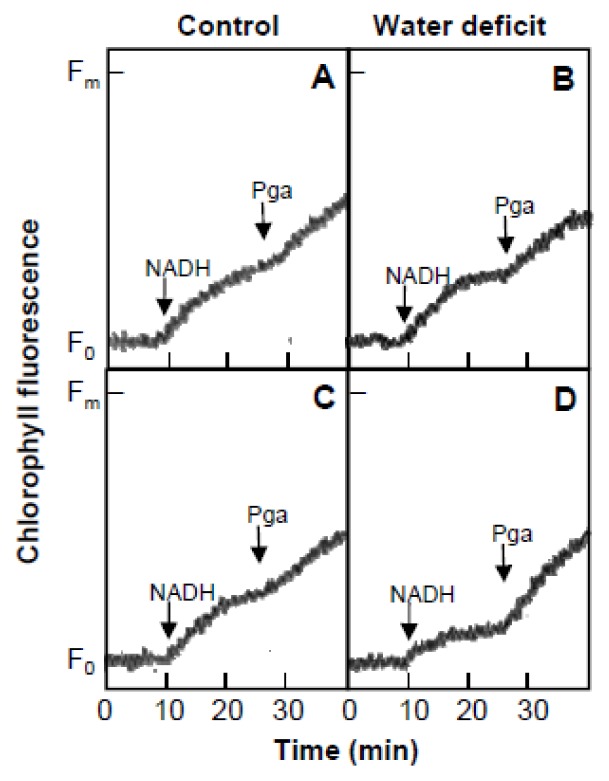
Effect of exogenous addition of NADH (2 mM) and *n*-propyl gallate (Pga, 1 mM) on the chlorophyll fluorescence level measured under low light in thylakoid membrane suspensions (50 μg Chl·mL^−1^) isolated from leaves recently detached (**A** and **B**) or incubated in water for 20 h under high light intensity (1400 μmol·m^−2^·s^−1^) at 24 °C (**C** and **D**) from *H. rosa-sinensis* plants in control conditions and after exposure to three photoperiods (18 h, 60 μmol·m^−2^·s^−1^ PPFD and 35 °C) with reduced irrigation (water deficit). The stress photoperiods were separated by 6 h night-periods at 24 °C. The figure shows typical curves from five independent replicates.

The PTOX activity in plastoquinone oxidation was assayed in recently isolated thylakoid membranes, as described by Joët *et al*. [46]. These authors observed that the addition of exogenous 2 mM NADH and the PTOX inhibitor 1 mM *n*-propyl gallate to thylakoid suspensions increased the apparent F_0_ chlorophyll fluorescence level measured under low non-actinic light, because the reduced state of the plastoquinone pool increased. The redox state of the plastoquinone pool was the result of competition between reduction by the NDH complex and oxidation by the PTOX. Chlorophyll fluorescence increased after the addition of 2 mM NADH and 1 mM *n*-propyl gallate under low measuring light (Figure 5) in thylakoid membrane suspensions (50 μg Chl·mL^−1^) isolated from the chloroplasts of leaves recently detached (Figure 5A,B) or incubated in water for 20 h under high light intensity (1400 μmol m^−2^ s^−1^) at 24 °C (Figure 5C,D). The thylakoids of leaves incubated under high light intensity from plants exposed to water deficit and heat (Figure 5D) showed a slower increase in NADH-induced chlorophyll fluorescence than the thylakoids of control leaves incubated under high light intensity and those of leaves recently detached from plants, both control and those exposed to water deficit and heat. The addition of *n*-propyl gallate significantly increased the chlorophyll fluorescence level in the thylakoids of leaves incubated under high light intensity from plants exposed to water deficit and heat, whereas the effect was slight in all other conditions, indicating higher PTOX activity in the leaves under high light intensity from plants with water deficit.

**Figure 6:**
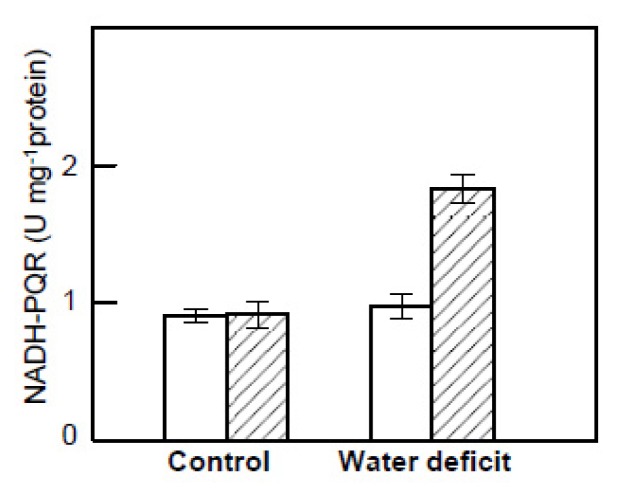
NADH-plastoquinone oxidoreductase (NADH-PQR) activity in the thylakoid membranes isolated from leaves recently detached (white bars) or incubated in water for 20 h under high light intensity (1400 μmol·m^−2^·s^−1^) at 24 °C (striped bars) from *H. rosa-sinensis* plants in control conditions and after exposure to three photoperiods (18 h, 60 μmol·m^−2^·s^−1^ PPFD and 35 °C) with reduced irrigation (water deficit). The stress photoperiods were separated by 6 h night-periods at 24 °C. The values are the means ± SE from three to four independent replicates.

The NADH dehydrogenase activity was assayed as NADH-plastoquinone oxidoreductase (NADH-PQR) activity in thylakoid membranes of leaves recently detached or incubated under high light intensity (Figure 6). Little difference in the activity was observed in leaves recently detached from control plants and those exposed to water deficit and heat. After incubation under high light, similar activity was observed in control plants, while in water deficit plants, the activity increased two-fold compared with the recently detached leaves.

## 3. Discussion

Water deficit and heat resulted in the downregulation of linear electron transport in leaves from *Hibiscus rosa-sinensis* plants, as indicated by a reduction in the photochemistry efficiency of PS II and in the capacity for electron transport. The quantum yield of PS II decreased with increasing photonic flux density, because of the accumulation of electrons on the PS II acceptor side. When light was not excessive, the PS II quantum yield kept its maximum value, and the relationship between the relative electron transport rate and the light intensity was linear (Figure 3, optimum line). The relative electron transport rate fell below the values predicted by the optimum line when the PS II quantum yield decreased due to excessive light. Eventually, a saturated rate was reached, which represents the photosynthetic electron transport capacity. This capacity, as well as the PS II quantum yields and the relative electron transport rates were lower in plants exposed to water deficit and heat than in control plants. However, the decrease of the maximal quantum yield of PS II (F_v_/F_m_) after stress photoperiods was very small, suggesting that chloroplasts are protected by mechanisms that dissipate excess excitation energy to prevent damage to the photosynthetic apparatus under adverse conditions. Regulating the flow of electrons through the photosynthetic electron transport chain is crucial to the health and survival of plants. The inhibition of photosynthetic linear electron transport, the major electron transfer pathway, could trigger alternative pathways, such as cyclic electron flow around PS I and chlororespiration [10]. Two parallel cyclic pathways exist around PS I [20], one sensitive to antimycin A, of which PGR5 is an essential component [21], and the other involving the NDH complex [20]. In this work, we observed that the incubation of leaves under high light intensity resulted in an increase in electron donation by NADPH and ferredoxin to plastoquinone, which was inhibited by antimycin A, in leaves of control plants, but not in leaves from plants exposed to water deficit and heat, suggesting that cyclic electron transport was stimulated by high light only in the control plants. In contrast, the activities of the chlororespiration enzymes, NDH complex and PTOX, increased after incubation under high light intensity in leaves from water deficit plants, but not from control plants, suggesting that chororespiration was stimulated in stressed plants.

Alternative routes to photosynthetic linear electron transport, such as cyclic electron flow around PS I and chlororespiratory pathways, may contribute to protecting the photosynthetic apparatus under stress conditions. It is known that cyclic electron flow increases in response to high light intensity [10,33], and the physiological relevance of NDH-mediated electron flow in chloroplasts under stress conditions has also been reported [22,35,39,41,42,44,47–50]. However, the cooperation between these pathways remains unclear [33]. The concerted action of NDH complex and PTOX would optimize the efficiency of the cyclic pathways, preventing over-reduction of the electron transfer chain [10,51] and reducing the accumulation of reactive oxygen species (ROS) by recycling electrons to the plastoquinone pool and, ultimately, to oxygen through PTOX, forming water in the stroma. Additionally, these pathways contribute to balancing ATP/NADPH requirements and to generating a large proton gradient and acidification of the lumen, that play a crucial role in the regulation, preventing the light-induced inactivation of both PS I and PS II through the formation of non-photochemical quenching [10,33].

The relative importance of chlororespiration and the cyclic electron pathways regulating the flow of electrons may differ in each plant species. We have described how the PGR5-dependent cyclic pathway is more active in sun plants, whereas in shade plants, other pathways involving the NDH complex and PTOX may be more important [44,50]. Additionally, stress conditions can also affect the relative importance of these alternative electron pathways regulating the flow of electrons in plants. In this work, we have seen that plants showed higher tolerance to high illumination when there was no water deficit. In such conditions, the NDH complex and PTOX activities did not increase, while the activity of electron donation by NADPH and ferredoxin to plastoquinone increased. However, in plants suffering a water deficit, the efficiency of PS II photochemistry decreased considerably and, under high illumination, the NDH complex and PTOX activities increased. The apparent correlation between the low functioning of PS II and the upregulation of PTOX and the thylakoidal NDH complex supports a role for chlororespiration in the protection against high light when other pathways, such as cyclic electron flow around PS I, are insufficient to protect PS II.

## 4. Experimental Section

### 4.1. Plant Material and Incubation Conditions

*Hibiscus rosa-sinensis* plants were grown in 500 mL pots at 22–25 °C in a greenhouse under natural light conditions (irradiation maxima of around 800 μmol m^−2^ s^−1^ PPFD) and controlled watering to avoid drought stress until flowering (control plants). For water deficit conditions, adult plants (0.4 m height) were transferred to cultivation chambers with 18 h photoperiods of white light of low intensity (60 μmol m^−2^ s^−1^ PPFD) supplied by 40W/10 Osram daylight fluorescent tubes (Augsburg, Germany) at 35 °C, followed by 6 h night-periods at 24 °C, decreasing the irrigation to 50 mL/day, which was applied after the start of the night period. For the high light intensity treatments, leaves were detached from control and water deficit plants after the third photoperiod and incubated for 20 h in Petri dishes containing water under white light of high intensity (1400 μmol m^−2^ s^−1^ PPFD) supplied by a 100 W Flood Osram lamp (Augsburg, Germany), at 24 °C. The experiments were replicated in five independent plants for each treatment.

### 4.2. Plant Water Status

Plant water status was estimated by measuring the relative water content of leaves (RWC). Leaves were collected and immediately weighed to determine fresh weight (FW). Leaves were then re-hydrated for 24 h at 4 °C in darkness to determine the turgid weight (TW) and subsequently oven-dried for 24 h at 85 °C to determine the dry weight (DW). The RWC was determined as 100 × (FW − DW)/(TW − DW).

### 4.3. Isolation of Thylakoid Membranes

Chloroplasts were isolated from leaves, as described by Quiles and Cuello [11], using an extraction buffer (pH 7.6) containing 0.35 M sucrose, 25 mM Na-HEPES, 2 mM Na_2_-EDTA, 2 mM ascorbic acid, 4 mM dithiothreitol, 10 mM MgCl_2_ and 1 mM phenylmethylsulfonyl fluoride. As reported previously, a comparison of cytochrome *c* oxidase-specific activity and polypeptide profiles in mitochondrial and chloroplast fractions indicated that the chloroplast preparation was essentially mitochondrion-free [52]. The chloroplasts were washed twice and osmotically broken with 10 mM Tricine, 10 mM NaCl and 10 mM MgCl_2_ (pH 7.8) buffer, as described previously [41]. The thylakoid membrane pellet was resuspended in buffer (pH 7.5) containing 200 mM sorbitol, 130 mM KCl and 5 mM potassium phosphate at a chlorophyll concentration of 0.4 mg mL^−1^, thus providing the thylakoid membrane suspension.

### 4.4. Chlorophyll Fluorescence Measurements

Chlorophyll fluorescence was measured in the thylakoid membrane suspension (50 μg Chl mL^−1^) using a PAM-210 chlorophyll fluorometer (Heinz Walz GmbH, Effeltrich, Germany), and chlorophyll fluorescence was imaged, using the MINI-version of the Imaging-PAM (Heinz Walz GmbH, Effeltrich, Germany) in entire leaves. Prior to the fluorescence measurements, the leaves were dark-adapted for 30 min. Minimal fluorescence yield (F_0_) was measured at a low frequency of pulse modulated measuring light, while maximal fluorescence yield (F_m_) was measured with the help of a saturation pulse. The maximal quantum yield of PS II (F_v_/F_m_) was calculated using the PAM fluorometer software (Heinz Walz GmbH: Effeltrich, Germany, 2006).

Light response curves were made by illuminating the entire leaves with actinic light of different intensities (60, 90, 120, 150, 210, 310, 440, 600, 850 and 1250 μmol m^−2^ s^−1^ PAR), with 2 min illumination periods at each intensity. After each illumination period, a saturation pulse was applied to determine the quantum yield of PS II and the relative electron transport rate, all of which were calculated using the PAM fluorometer software.

### 4.5. NADH-Plastoquinone Oxidoreductase Activity

The NADH-plastoquinone oxidoreductase (NADH-PQR) activity was determined, as described by Gamboa *et al.*[50], by measuring NADH oxidation at 340 nm in a Perkin Elmer (Germany) spectrophotometer at 25 °C. One unit (U) of enzymatic activity is defined as the amount of enzyme preparation that oxidized 1 μmol of substrate (NADH) per minute in the reaction conditions. The extinction coefficient of 6.22 mM^−1^ cm^−1^ at 340 nm was used to calculate the NADH oxidation rate.

### 4.6. Other Determinations

Protein was quantified using the method of Lowry *et al.*[53] after precipitation with 10% (*w*/*v*) trichloroacetic acid. Chlorophyll was determined by Lichtenthaler and Wellburn’s [54] method using 80% (*v*/*v*) acetone as solvent. Densitometric analysis and estimation of the polypeptide molecular masses were performed by an ACTIB 1D digital image analyzer (Microptic, Barcelona, Spain).

## 5. Conclusions

The relative importance of chlororespiration and the cyclic electron pathways in the tolerance of photosynthesis to high illumination differs under stress conditions. When plants are not subjected to stress, the contribution of chlororespiration to regulating photosynthetic electron flow is not relevant, and another pathway, such as the ferredoxin-mediated cyclic pathway, is more important. However, when PS II activity is inhibited by water deficit and heat, chlororespiration, together with other routes of electron input to the electron transfer chain, is probably essential, not only to support cyclic electron flow and ATP production in chloroplasts, but also to reduce the formation of ROS by preventing over-reduction of the plastoquinone pool in the chloroplasts.
